# Risk of Urological Cancer Among Boys and Men Born with Hypospadias: A Swedish Population-based Study

**DOI:** 10.1016/j.euros.2023.09.009

**Published:** 2023-09-28

**Authors:** Lottie Phillips, Cecilia Lundholm, Catarina Almqvist, Anna Skarin Nordenvall, Agneta Nordenskjöld

**Affiliations:** a Department of Women’s and Children’s Health and Center of Molecular Medicine, Karolinska Institutet, Stockholm, Sweden; b Deparment of Medical Epidemiology and Biostatistics, Karolinska Institutet, Stockholm, Sweden; c Pediatric Allergy and Pulmonology Unit, Astrid Lindgren Children’s Hospital, Karolinska University Hospital, Stockholm, Sweden; d Department of Radiology, Karolinska University Hospital, Stockholm, Sweden; e Department of Pediatric Surgery, Astrid Lindgren Children’s Hospital, Karolinska University Hospital, Stockholm, Sweden

**Keywords:** Cancer, Cohort design, Hypospadias, Urology

## Abstract

**Background:**

Hypospadias is a common genital malformation among boys. Studies indicate that hypospadias is associated with a higher risk of testicular cancer. Other forms of urological cancer may be linked to hypospadias via a mutual aetiology, hormonal dysfunction, or hypospadias complications, but this has not yet been studied.

**Objective:**

To investigate the association between hypospadias and testicular cancer and the risk of other urological cancers among individuals born with hypospadias.

**Design, setting, and participants:**

The study used a population-based male cohort born in Sweden in 1964–2018. Exposure was hypospadias diagnosis in national registers. Outcomes were defined using the Swedish Cancer Register. An extended cohort born from 1940 was used to study cancers among older men. Biological brothers and fathers were linked to investigate familial coaggregation.

**Outcome measurements and statistical analysis:**

Associations were assessed using Cox proportional-hazards regression analysis, with results presented as hazard ratios.

**Results and limitations:**

We found that hypospadias was associated with a higher risk of testicular cancer (hazard ratio 2.04, 95% confidence interval 1.42–2.92), especially for proximal hypospadias, but did not observe any clear familial coaggregation of hypospadias and testicular cancer. Hypospadias was associated with Wilms’ tumour in childhood. We also found an association between hypospadias and bladder and urethral cancers, but not prostate cancer. The number of cases with hypospadias was small and the results for cancers among older men may be impacted by limitations in register coverage.

**Conclusions:**

Our study supports the hypothesis of a higher risk of testicular cancer for men with hypospadias, especially with proximal phenotypes. Hypospadias may also be associated with a higher risk of lower urinary tract cancers, although this requires further investigation in older cohorts.

**Patient summary:**

Boys and men in whom the opening of the urethra is not at the end of the penis (called hypospadias) at birth are at higher risk of developing testicular cancer, although their overall risk is still low. They may also have a higher risk of developing other forms of cancer in the urinary tract.

## Take Home Message

Men with hypospadias have a higher (albeit still low) risk of testicular cancer that is not clearly shared by family members. Hypospadias may also be associated with urethra and bladder cancers, but this possibility requires further investigation, especially in older cohorts.

## 1 Introduction

Hypospadias is a common congenital malformation in boys [1]. At gestational weeks 8–15, the formation of the external genitalia is disrupted in this condition, leading to incomplete development of the urethra and foreskin, sometimes with penile curvature. The urethral meatus is located proximal to the tip of the glans, across a spectrum of phenotypes. Hypospadias aetiology involves genetic and environmental factors and it is hypothesised that the condition is often related to impaired androgen function [2]. Treatment is surgical, typically in early childhood. Complications include the development of urethral fistulas or strictures, sometimes years after the primary surgery [3]. Hypospadias can also affect later life in other ways, including sexual and reproductive health [4], [5], [6]. The risk of different outcomes is often greater for those with proximal hypospadias, and can further differ according to treatment, aetiological factors, and comorbidities. Despite the occurrence of delayed negative outcomes, few studies have looked at middle-aged and older men [6], [7].

**Fig. 1:**
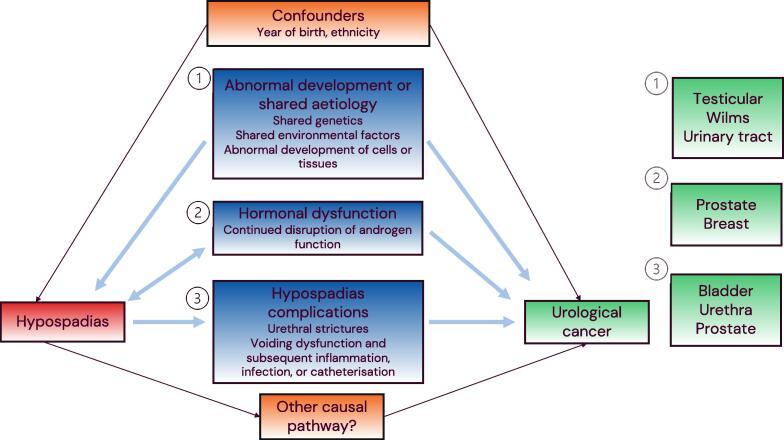
Schematic overview of the research question and underlying hypotheses regarding associations between hypospadias and urological cancers. Types of urological cancers that may be primarily linked to each hypothesis (1–3) are shown on the right.

There are a few reasons why hypospadias may be related to the risk of urological cancer (Fig. 1). First, cancer and hypospadias may both result from abnormal development or shared genetic or environmental aetiological factors. Studies have previously found links between body-site malformations and cancer [8], [9]. Given the embryology and genetics of hypospadias, the risk of Wilms’ tumour and other urinary tract cancers may be higher, while it has been hypothesised that both testicular cancer and hypospadias result from abnormal development of the testes [2], [10].

Second, the effects of continuous low endogenous androgen may impact cancer risk. A few studies have found that hormone profiles are affected in adults with proximal hypospadias in particular. Hypospadias also appears to be associated with androgen-related morbidity, including hypogonadism and cardiometabolic disease [5], [11], [12], [13], [14]. Both prostate and male breast cancers are biologically related to androgen and oestrogen function, although it is not certain if endogenous testosterone impacts prostate cancer risk [15], [16], [17].

Finally, cancer risk may be a consequence of hypospadias outcomes. Several hypospadias complications cause voiding dysfunction, which can result in inflammation, infections, and catheterisation of the bladder [6]. Studies have shown associations between these factors and prostate and bladder cancers, while urethral cancer is related to a history of urethral stricture [18], [19], [20], [21], [22].

While results from a few published studies have indicated a higher risk of testicular cancer for men with hypospadias, the risk of other urological cancers remains unknown [23], [24], [25]. To address this knowledge gap, we investigated the association between hypospadias and testicular cancer, including possible familial coaggregation, and other forms of urological cancer.

## 2 Patients and methods

**Fig. 2:**
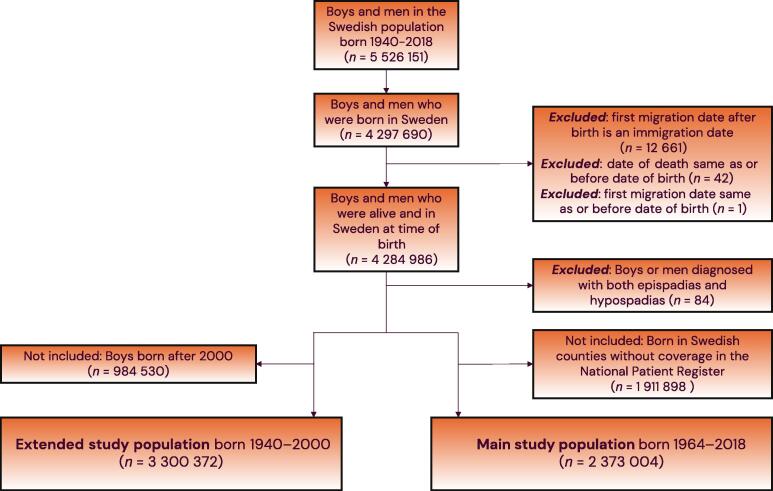
Flowchart showing individuals included in and excluded from the study cohort from boys and men in the Swedish population.

This study is reported in accordance with the STROBE guidelines for observational studies. Ethical permission was granted by the Swedish Ethical Review Authority. We used a cohort study design and a cohort consisting of men born in Sweden. Men with a diagnosis of both hypospadias and epispadias were excluded owing to the uncertainty for diagnosis (Fig. 2).

### 2.1 National registers

Sweden has multiple national registers that are extensively used in research. Each Swedish resident has a personal identification number that can be used to link data across sources [26]. All demographic data in this study (place of birth, dates of birth, death, and migration, registered parents and brothers, and parents’ region of origin) were taken from registers maintained by Statistics Sweden. Data were pseudonymised before being delivered to the researchers. The National Board of Health and Welfare maintains the National Patient Register (NPR), which started registering inpatient care in 1964, day surgery in 1997, and outpatient specialist care in 2001, and the Medical Birth Register (MBR), which has registered prenatal and perinatal data since 1973 [27], [28]. Data are retrieved semi-automatically from medical records, including diagnoses defined using International Classification of Diseases (ICD) codes. The Swedish Cancer Register (SCR) has data on all cancers histopathologically verified nationally since 1958. Cancer types are classified according to both anatomic locations (ICD codes) and morphological type (ICD-O).

### 2.2 Exposure and outcomes

The study exposure was hypospadias, classified using ICD-7 to ICD-10. Any individual diagnosed at least once in the NPR or the MBR was defined as having hypospadias. Specific codes were used in ICD-8 and ICD-10 to separate distal (penile and glandular) and proximal (penoscrotal and perineal) hypospadias (Supplementary Table 1). As diagnoses can be registered for health care visits years after birth, individuals identified as having distal or proximal hypospadias could be born throughout the study period, despite limitations in ICD-7 and ICD-9.

Cancer outcomes were taken from the SCR. Cancer types were classified by location (breast, upper urinary tract, bladder, urethra, prostate, testes, and other male reproductive organs as defined in Supplementary Table 2). Testicular cancer and Wilms’ tumour were further specified using morphological data (Supplementary Table 3).

### 2.3 Covariates

Year of birth was included as a confounder, as hypospadias prevalence, hypospadias surgery, and cancer incidence have changed over time [1]. We used maternal country of birth as a proxy to adjust for confounding by ethnicity (Table 1). Cryptorchidism was defined using both diagnosis and surgical treatment data from the NPR to limit misclassification (Supplementary Table 1).

**Table 1:** Characteristics of the study population born in 1940–2018: unadjusted frequencies

Parameter	Individuals, *n* (%)			
	No hypospadias	Any hypospadias	Distal hypospadias a	Proximal hypospadias a
Individuals (*N*)	4 267 353	17 549	12 205	1469
Birth year				
1940–1951	691 188 (16)	301 (1.7)	97 (0.8)	11 (0.8)
1952–1963	632 950 (15)	446 (2.5)	188 (1.5)	28 (1.9)
1964–1975	686 421 (16)	1862 (11)	1 276 (10)	98 (6.7)
1976–1987	583 405 (14)	2 679 (15)	1 862 (15)	106 (7.2)
1988–1999	653 450 (15)	3819 (22)	1949 (16)	303 (21)
2000–2018	1 019 939 (24)	8442 (48)	6833 (56)	923 (63)
Mother’s country of birth				
Nordic country (including Sweden)	3 841 085 (90)	14 243 (81)	9750 (80)	1113 (77)
Greater Europe	160 442 (3.8)	1221 (7.0)	879 (7.2)	100 (6.8)
Africa	48 686 (1.1)	505 (2.9)	383 (3.1)	76 (5.2)
Asia	126 341 (3.0)	1316 (7.5)	1 016 (8.3)	137 (9.3)
Other	34 495 (0.8)	156 (0.9)	108 (0.9)	15 (1.0)
Data missing	56 304 (1.3)	108 (0.6)	69 (0.6)	8 (0.5)
Breast cancer	479 (0.0)	NA	NA	NA
Upper urinary tract cancer b	9132 (0.2)	NA	NA	NA
Bladder cancer	14 634 (0.3)	15 (0.1)	6 (0.1)	NA
Urethral cancer	153 (0.0)	5 (0.0)	NA	NA
Prostate cancer	96 636 (2.3)	42 (0.2)	11 (0.1)	NA
Other male reproductive cancer	2147 (0.1)	NA	NA	NA
**Individuals born after 1964**^c^				
Cryptorchidism	28 882 (1.2)	695 (4.6)	332 (3.1)	232 (17)
Wilms’ tumour	167 (0.0)	5 (0.0)	NA	NA
Testicular cancer	3299 (0.1)	32 (0.2)	21 (0.2)	9 (0.7)
Seminoma	1425 (0.1)	19 (0.1)	11 (0.1)	7 (0.6)
Nonseminoma	1249 (0.1)	6 (0.0)	5 (0.1)	NA
Paediatric testicular cancer d	178 (0.0)	6 (0.0)	NA	NA

NA = number not presented for categories with *n* < 5.

a Distal hypospadias (70% of hypospadias cases) and proximal hypospadias (8.4% of hypospadias cases) phenotypes were identified according to phenotype-specific codes in ICD-8 and ICD-10 (Supplementary Table 1). These results do not sum to 100% of men with hypospadias, as individuals could have received an unspecific diagnosis (Section 2.2).

b Kidney, kidney pelvis, and ureter cancer, excluding all kidney cancer cases diagnosed before the age of 18 yr.

c The main study population born in 1964–2018 in a Swedish county with full coverage in the National Patient Register.

d Testicular cancer diagnosed before the age of 18 yr.

### 2.4 Statistical analysis

Cox proportional-hazards regression analysis was used to assess associations between hypospadias and the outcomes (Wilms’ tumour and testicular, bladder, urethral, and prostate cancers) with attained age as the underlying timescale. Time-to-event analysis was selected given variations in follow-up time between individuals. When numbers were sufficient, results for distal hypospadias and proximal hypospadias groups, as well as “any hypospadias”, were compared to the group without hypospadias. Individuals were censored at date of migration, date of death, or the end of follow-up on the December 31, 2018. Multiple Cox regression analysis was used to adjust for confounding. Models were tested for proportional hazards using Schoenfeld’s residuals and stratified by maternal birthplace when testing indicated that hazards were not proportional. Birth year was included as a continuous covariate to avoid potential issues stemming from stratification by birth-year categories.

To investigate the risk of testicular cancer and Wilms’ tumour, boys and men born in 1964–2018 in a Swedish county with coverage in the NPR (started in 1964) were followed from birth. Individuals were only considered at risk of Wilms’ tumour up to the age of 18 yr, after which they were censored. For testicular cancer, we performed three sensitivity analyses: (1) exclusion of individuals with cryptorchidism, which is associated with hypospadias and a primary risk factor for testicular cancer [29]; (2) use of a stricter definition of seminoma to assess whether different outcome definitions yield different results; and (3) exclusion of individuals born during the years for ICD-7 (1958–1968) and ICD-9 (1987–1996) to test for bias introduced by the lack of phenotype-specific codes during these time periods.

To investigate whether the association between hypospadias and testicular cancer is impacted by shared familial factors, we conducted a familial coaggregation for the two conditions. All full- and half-brother pairs in the study population were identified. To assess whether restriction of the study population to brothers introduced bias, we first measured the association between hypospadias and testicular cancer within the same individual. We also tested for interaction between brother status (with vs without a full or half brother) and hypospadias status in the full cohort. Given the absence of significant interaction, for every individual (person A) in the cohort of brothers, we defined the exposure as their brother’s (person B) hypospadias status, with testicular cancer in person A as the outcome. Multiple births were excluded from the study population. Cluster-robust standard errors were used to account for clustering of brother pairs (ie, multiple brothers) within families. We controlled for hypospadias status and birth year in person A [30]. To further understand individual and familial factors, we included models with an interaction between hypospadias status in person A and in person B. An equivalent analysis was performed for father-son pairs using the biological son’s exposure status and testicular cancer in the father.

To allow for longer follow-up in an older population when studying urinary tract and prostate cancers, these analyses were performed in an extended cohort of men born in 1940–2000, with follow-up starting from the age of 18 yr. For men born before register coverage, if the first hypospadias diagnosis was recorded in the registers in conjunction with their cancer diagnosis, we could estimate a falsely high proportion of men with hypospadias and cancer. To address this, a sensitivity analysis was performed for all outcomes in the extended cohort excluding those diagnosed with hypospadias for the first time in our data within 1 yr before or after their registered cancer diagnosis.

All statistical analyses were performed using Stata version 17.0 (Stata Corporation, College Station, TS, USA).

## 3 Results

A total of 4 284 902 men born in Sweden in 1940–2018 comprised the full study population, including 17 549 men defined as having hypospadias. Of the men included in the main study population, 2 373 004 were born in 1964–2018 (Fig. 2). Among boys aged <18 yr there were cases of testicular cancer and Wilms’ tumour, but very few cases of other urological cancers. The number of individuals with both hypospadias and breast cancer, other male reproductive cancers (penis, epididymis, and scrotum), or upper urinary tract cancers (not including paediatric kidney cancer) was less than five, and therefore no further analysis was conducted for these categories (Table 1).

**Fig. 3:**
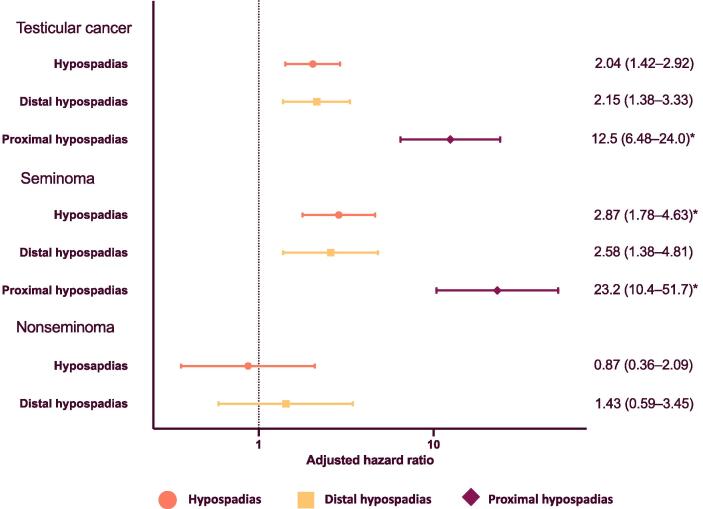
Testicular cancer among boys and men born with hypospadias in 1964–2018. Adjusted hazards ratios for the associations between hypospadias and testicular cancer. Individuals were followed from birth until death, migration, diagnosis of testicular cancer, or the end of the study period (31 December, 2018), whichever occurred first. All analyses were adjusted for year of birth and mother’s place of birth as a proxy for ethnicity. Hazard ratios for distal hypospadias and for proximal hypospadias are in comparison to men without hypospadias. * Hazards were not fully proportional across the follow-up period. The effect estimates should be interpreted as an average over time.

Follow-up for our main study population (born in 1964–2018) from birth revealed a significant association between hypospadias and testicular cancer (hazard ratio [HR] 2.04, 95% confidence interval [CI] 1.42–2.92; Fig. 3; unadjusted data in Supplementary Table 4). The association was stronger for men with proximal hypospadias. Most cases of testicular cancer in the groups with and without hypospadias were diagnosed in adulthood (Table 1). Exclusion of individuals with cryptorchidism had little impact on the effect estimates (Supplementary Table 5). We found a significant and somewhat stronger association when looking at seminoma specifically, and no clear association with nonseminoma (Fig. 3). A sensitivity analysis using a stricter definition of seminoma gave similar results (Supplementary Table 7). For any testicular cancer and for just seminoma, the results for distal and proximal hypospadias were very similar after excluding individuals born during the time periods for ICD-7 and ICD-9, when phenotype-specific codes were lacking (Supplementary Table 8).

**Fig. 4:**
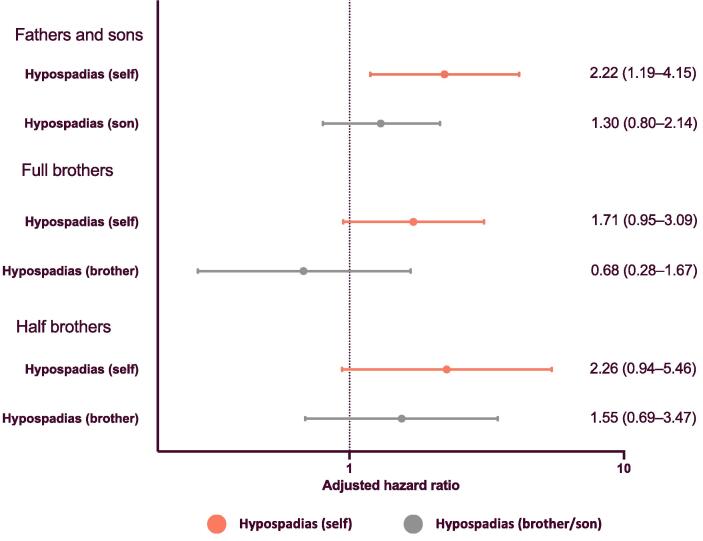
Familial coaggregation of testicular cancer and hypospadias for males born in 1964–2018. Populations were restricted to only fathers and sons, only full brothers, and only half brothers for diagnosis of testicular cancer as the outcome. The association between hypospadias and cancer within an individual (person A) was first tested (self). Then the association with the relative’s (person B) diagnosis of hypospadias, adjusted for exposure status and birth year, was assessed for person A. All multiple births were excluded from analyses of brothers. Cluster robust standard errors were used to account for clustering within families. Individuals were followed from birth until death, migration, diagnosis of testicular cancer, or the end of the study period (December 31, 2018), whichever occurred first.

We found that restricting the main study population to only full brothers somewhat skewed our estimate for the association between hypospadias and testicular cancer towards the null hypothesis (Fig. 4), and in the main population the HR was lower for those with at least one full brother, although there was no statistically significant difference between men with and without full brothers (Supplementary Table 9). However, restriction of the population to fathers and sons did not impact the estimate. Looking at familial coaggregation, we found no evidence of an association between the incidence of testicular cancer and having a son, full brother, or half brother with hypospadias (Fig. 4; unadjusted data in Supplementary Table 10). Testing for interaction, we still found no evidence of higher testicular cancer risk for individuals with a family member with hypospadias. For full brothers, the results indicate that individuals with hypospadias whose brother also has hypospadias may have a particularly high risk of testicular cancer (Supplementary Table 11).

In the main study population, we found an association between hypospadias and Wilms’ tumour (HR 4.21, 95% CI 1.73–10.3, followed to age 18 yr).

**Fig. 5:**
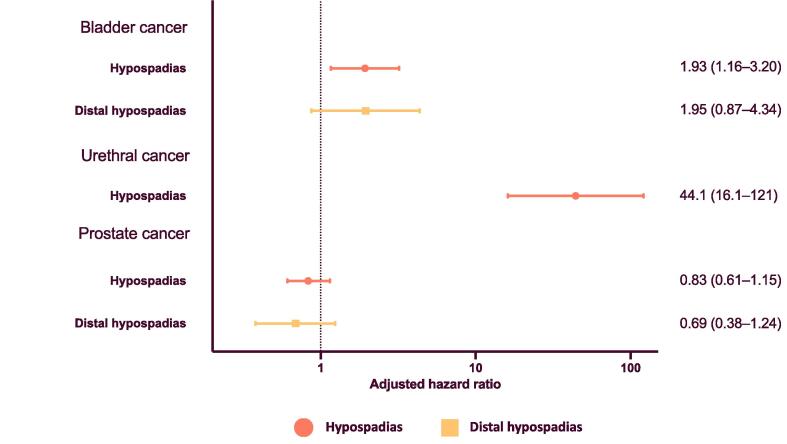
Association between hypospadias and cancers of the lower urinary tract and prostate for men born in 1940–2000. Individuals were followed from the age of 18 yr until death, migration, diagnosis of cancer, or the end of the study period (December 31, 2018), whichever occurred first. All analyses are adjusted for year of birth and mother’s place of birth as a proxy for ethnicity. Hazard ratios for distal hypospadias are in comparison to men without hypospadias.

In the extended cohort born from 1940, follow-up was from age 18 yr to a maximum age of 79 yr, with a median time at risk for individuals without an event (bladder, urethral, or prostate cancer) of 29 yr in the whole population and 14 yr for those with hypospadias. We found an association between hypospadias and bladder cancer (HR 1.93, 95% CI 1.16–3.20) and urethral cancer (HR 44.1, 95% CI 16.1–121) (cases with urethral cancer and hypospadias included both urothelial and squamous cell carcinoma), but there was no clear association between hypospadias and prostate cancer (HR 0.83, 95% CI 0.61–1.15; Fig. 5). The effect estimates were partly reduced for all associations in the sensitivity analysis that excluded men with a first hypospadias diagnosis registered within 365 d of their cancer diagnosis. For bladder cancer, the estimate was no longer statistically significant (Supplementary Fig. 1).

## 4 Discussion

We used national Swedish registers in a population-based study to investigate the risk of urological cancer for boys and men born with hypospadias on the basis of three underlying hypotheses (Fig. 1). Regarding the hypothesis of a shared aetiology, we found an association between hypospadias and testicular cancer within the individual that increased with the severity of the hypospadias phenotype, but no familial coaggregation within full-brother, half-brother, or father-and-son pairs. We also found that hypospadias was associated with a higher risk of Wilms’ tumour. Regarding our hypotheses on hormone dysfunction or the consequences of hypospadias complications, we found a higher risk of bladder and urethral cancers, but no clear association with prostate cancer. There were too few hypospadias cases with adult upper urinary tract cancer, breast cancer, or other male reproductive cancers for further analysis.

Our effect estimate for the risk of testicular cancer in individuals with hypospadias is in line with previous research [23], [25]. Specifically, a Swedish case-control study using the SCR found an association between hypospadias or epispadias and testicular germ cell cancer after controlling for cryptorchidism (odds ratio [OR] 2.25, 95% CI 1.17–4.32). The study revealed a strong association between hypospadias or epispadias and seminoma (OR 6.11, 95% CI 2.53–14.75), but not nonseminoma (OR 0.75, 95% CI 0.22–2.52) [25]. Our results for nonseminoma were similar, while we had a lower effect estimate for seminoma, with confidence intervals that overlap with those in the case-control study. We also found a relatively higher risk for men with proximal hypospadias, which has not previously been studied to the best of our knowledge. Given that proximal hypospadias is a more severe phenotype, this finding supports the biological plausibility of our results. It may indicate a greater impact on the development of the testes, or a higher degree of, or different, shared aetiological factors. Exclusion of men with co-occurrence of cryptorchidism had no significant impact on our estimates, supporting the previous finding of an independent association [23], [25].

We found no clear familial coaggregation of hypospadias and testicular cancer, in accordance with Schnack et al [23]. The association between hypospadias and testicular cancer was somewhat reduced when the analysis was restricted to a population consisting only of full brothers, which may indicate selection bias. This is further supported by our finding of a lower HR for testicular cancer for individuals with a full brother than for those without full brothers, although the difference was not statistically significant. If there are familial factors that link the two conditions, they may also reduce fertility, which would reduce the likelihood of a father having multiple sons [31]. This bias should be considered in family-based study designs looking at these and related conditions. We also found no evidence of familial coaggregation in fathers and sons, which was not impacted by selection in the same way. In summary, individual factors in those with hypospadias appear to play an important role in testicular cancer risk. However, we cannot exclude the possibility that familial factors may also play an important role, and it is possible that those with familial and sporadic hypospadias have different risk profiles.

We found a significant association between hypospadias and the risk of Wilms’ tumour, although the overall risk was very low. Hypospadias and Wilms’ tumour have been described together in genetic syndromes such as those resulting from mutations in the *WT1* gene [8]. Our results probably reflect cases with genetic overlap, although we lack data to confirm this.

We hypothesised that cancer in the urinary tract could result from hypospadias complications, although abnormal development may also play a role. If the associations observed do result more causally from hypospadias, efforts to improve hypospadias outcomes may help in reducing cancer risk. The strongest association we found was with urethral cancer, although the absolute risk was very low, with only five cases reported for patients with hypospadias. Urethral cancer is strongly associated with urethral stricture, and it is thought that chronic inflammation plays a key role in the pathogenesis, making an association with hypospadias biologically plausible [22]. Further clinical research detailing cases of urethral cancer in males born with hypospadias could help in elucidating this rare outcome.

Finally, we found no clear association between hypospadias and prostate cancer. If low endogenous testosterone is protective, men with hypospadias may have a lower risk of developing prostate cancer. However, pooled estimates from observational studies have not been able to find a protective effect [16]. Conversely, there has been concern for decades that testosterone treatment, which is more common for men with hypospadias, may increase cancer risk, although there is no clear evidence to support this [5], [32]. There is also increasing evidence of a link between prostate cancer and inflammation, which may indicate a positive association with hypospadias [33]. While our results may reflect a true absence of any association, or separate associations working in different directions, they may also be a consequence of limited follow-up time [34].

### 4.1 Strengths and limitations

Our large, population-based study investigated rare cancer outcomes using the SCR, which has national coverage and high validity. As pathological confirmation is needed, sensitivity is generally lower for older patients and for cancer types that are often not biopsied or surgically removed. In a study assessing completeness, the highest sensitivity was observed for urological cancers overall [35].

In general, our results should not be impacted by surveillance bias, as there is no current screening programme for cancer for patients with hypospadias, and many patients with hypospadias are not followed past childhood [36]. However, some cases of prostate cancer may be incidentally diagnosed in men with hypospadias seeking help for voiding dysfunction or urinary retention, resulting in differential misclassification. This could mean that the risk of prostate cancer was overestimated.

As most of the men in our study population with confirmed hypospadias were young, the mean follow-up is relatively short and may have been insufficient to measure true associations with cancers strongly associated with age, including urinary tract cancers and especially prostate cancer. Our results are instead more a reflection of the HR for early-onset cases. The short mean follow-up also limited the number of cancer outcomes, and any association with urethral cancer in particular is preliminary given the very low number of cases in the hypospadias cohort.

As data on diagnosis of hypospadias are only available from 1964, there is also some concern that for the oldest cohort in the study population (born from 1940) we would identify men who received a reported hypospadias diagnosis in conjunction with their cancer diagnosis, while others might go undetected. Our sensitivity analyses excluding those with a diagnosis first registered within 365 d of their cancer diagnosis reduced our effect estimates to some degree, indicating that there may be some bias, although the low number of cancer cases in the hypospadias cohort also limited the statistical power.

Finally, as mentioned in the Introduction, cancer risk may differ because of factors such as phenotype, underlying genetic or environmental factors, and treatment. When possible, we looked at the different hypospadias phenotypes separately, while further specification is not possible owing to small numbers and limitations in the registers. We also cannot confirm the mechanisms underlying these associations, and noncausal pathways other than those hypothesised in Figure 1 may play a role. Further research is needed for a fuller understanding of which individuals with hypospadias are at risk of developing cancer and why. Our findings for urinary tract and prostate cancers in particular should be interpreted in relation to these limitations.

## 5 Conclusions

Our results support previous research demonstrating a higher risk of testicular cancer for boys and men born with hypospadias and is the first to show a stronger association for proximal hypospadias. However, we could not find evidence of familial coaggregation of the two conditions, indicating that individual factors in those with hypospadias are important for testicular cancer risk. We also found a higher risk of Wilms’ tumour for boys born with hypospadias. Finally, our results indicate a higher risk of lower urinary tract cancers in adult men, but no association with prostate cancer.

It is important to highlight that the absolute risk of developing these cancer types among individuals with hypospadias was low and probably depends on the hypospadias phenotype and other factors. In particular, the novel results for lower urinary tract cancer are too preliminary to draw conclusions regarding specific clinical implications at this point. We recommend increased awareness of the risks associated with hypospadias and further investigation, including studies in older cohorts, to identify and optimise care for individuals at risk with greater precision.

  ***Author contributions***: Lottie Phillips had full access to all the data in the study and takes responsibility for the integrity of the data and the accuracy of the data analysis.

  *Study concept and design*: Phillips, Skarin Nordenvall, Nordenskjöld.

*Acquisition of data*: Phillips, Skarin Nordenvall, Nordenskjöld, Almqvist.

*Analysis and interpretation of data*: Phillips, Lundholm, Skarin Nordenvall, Nordenskjöld.

*Drafting of the manuscript*: Phillips.

*Critical revision of the manuscript for important intellectual content*: Phillips, Skarin Nordenvall, Nordenskjöld, Almqvist, Lundholm.

*Statistical analysis*: Phillips, Lundholm, Skarin Nordenvall.

*Obtaining funding*: Phillips, Nordenskjöld.

*Administrative, technical, or material support*: Lundholm, Almqvist, Nordenskjöld.

*Supervision*: Lundholm, Almqvist, Skarin Nordenvall, Nordenskjöld.

*Other*: None.

  ***Financial disclosures:*** Lottie Phillips certifies that all conflicts of interest, including specific financial interests and relationships and affiliations relevant to the subject matter or materials discussed in the manuscript (eg, employment/affiliation, grants or funding, consultancies, honoraria, stock ownership or options, expert testimony, royalties, or patents filed, received, or pending), are the following: None.

  ***Funding/Support and role of the sponsor*:** This research was financially supported by the Swedish Research Council through grant 2016- 01642. Further financial support was received from Foundation Frimurare Barnhuset, Sällskapet Barnavård, HKH Kronprinsessan Lovisas förening för barnasjukvård, Stockholm Regional Council, and the Clinical Scientist Training Programme and Research Internship at Karolinska Institutet. The study funding was used to support data collection and administration, and the sponsors played no direct role in the study.

## References

[1] Skarin Nordenvall A., Frisén L., Nordenström A., Lichtenstein P., Nordenskjöld A. (2014). Population based nationwide study of hypospadias in Sweden, 1973 to 2009: incidence and risk factors. J Urol.

[2] van der Zanden L.F.M., van Rooij I.A.L.M., Feitz W.F.J.J., Franke B., Knoers N.V.A.M., Roeleveld N. (2012). Aetiology of hypospadias: a systematic review of genes and environment. Hum Reprod Update.

[3] Snodgrass W., Bush N. (2022). Do new complications develop during puberty after childhood hypospadias repair?. J Urol.

[4] Skarin Nordenvall A., Chen Q., Norrby C. (2020). Fertility in adult men born with hypospadias: a nationwide register-based cohort study on birthrates, the use of assisted reproductive technologies and infertility. Andrology.

[5] Phillips L., Lundholm C., Kvist U., Almqvist C., Nordenskjöld A., Skarin N.A. (2022). Increased androgen-related comorbidity in adolescents and adults born with hypospadias: a population-based study. Andrology.

[6] Leunbach T.L., O’Toole S., Springer A., Williamson P.R., Ahmed S.F. (2020). A systematic review of core outcomes for hypospadias surgery. Sex Dev.

[7] O’Kelly F., Nason G.J., McLoughlin L.C., Flood H.D., Thornhill J.A. (2015). A comparative bibliometric analysis of the top 150 cited papers in hypospadiology (1945–2013). J Pediatr Urol.

[8] Bleeker F.E., Hopman S.M., Hennekam R.C. (2014). Co-occurrence in body site of malformations and cancer. Eur J Med Genet.

[9] Carozza S.E., Langlois P.H., Miller E.A., Canfield M. (2012). Are children with birth defects at higher risk of childhood cancers?. Am J Epidemiol.

[10] Boisen K.A., Main K.M., Rajpert-De Meyts E., Skakkebaek N.E. (2006). Are male reproductive disorders a common entity?. Ann N Y Acad Sci.

[11] Kumar S., Tomar V., Yadav S.S., Priyadarshi S., Vyas N., Agarwal N. (2016). Fertility potential in adult hypospadias. J Clin Diagn Res.

[12] Asklund C., Jensen T.K., Main K.M., Sobotka T., Skakkebæk N.E., Jørgensen N. (2010). Semen quality, reproductive hormones and fertility of men operated for hypospadias. Int J Androl.

[13] Moriya K., Mitsui T., Tanaka H., Nakamura M., Nonomura K. (2010). Long-term outcome of pituitary-gonadal axis and gonadal growth in patients with hypospadias at puberty. J Urol.

[14] Lucas-Herald A.K., Montezano A.C., Alves-Lopes R. (2022 May 14). Vascular dysfunction and increased cardiovascular risk in hypospadias. Eur Heart J..

[15] Ruddy K.J., Winer E.P. (2013). Male breast cancer: risk factors, biology, diagnosis, treatment, and survivorship. Ann Oncol.

[16] Boyle P., Koechlin A., Bota M. (2016). Endogenous and exogenous testosterone and the risk of prostate cancer and increased prostate-specific antigen (PSA) level: a meta-analysis. BJU Int.

[17] Klap J., Schmid M., Loughlin K.R. (2015). The relationship between total testosterone levels and prostate cancer: A review of the continuing controversy. J Urol.

[18] Reid S., Brocksom J., Hamid R. (2021). British Association of Urological Surgeons (BAUS) and Nurses (BAUN) consensus document: management of the complications of long-term indwelling catheters. BJU Int.

[19] Hird A.E., Saskin R., Liu Y. (2021). Association between chronic bladder catheterisation and bladder cancer incidence and mortality: a population-based retrospective cohort study in Ontario, Canada. BMJ Open.

[20] Bayne C.E., Farah D., Herbst K.W., Hsieh M.H. (2018). Role of urinary tract infection in bladder cancer: a systematic review and meta-analysis. World J Urol.

[21] Sfanos K.S., Yegnasubramanian S., Nelson W.G., De Marzo A.M. (2018). The inflammatory microenvironment and microbiome in prostate cancer development. Nat Rev Urol.

[22] Grivas P.D., Davenport M., Montie J.E., Kunju L.P., Feng F., Weizer A.Z. (2012). Urethral cancer. Hematol Oncol Clin North Am.

[23] Schnack T.H., Poulsen G., Myrup C., Wohlfahrt J., Melbye M. (2010). Familial coaggregation of cryptorchidism, hypospadias, and testicular germ cell cancer: a nationwide cohort study. J Natl Cancer Inst.

[24] Schneuer F.J., Milne E., Jamieson S.E. (2018). Association between male genital anomalies and adult male reproductive disorders: a population-based data linkage study spanning more than 40 years. Lancet Child Adolesc Health.

[25] Trabert B., Zugna D., Richiardi L., McGlynn K.A., Akre O. (2013). Congenital malformations and testicular germ cell tumors. Int J Cancer.

[26] Ludvigsson J.F., Otterblad-Olausson P., Pettersson B.U., Ekbom A. (2009). The Swedish personal identity number: possibilities and pitfalls in healthcare and medical research. Eur J Epidemiol.

[27] Ludvigsson J.F., Andersson E., Ekbom A. (2011). External review and validation of the Swedish national inpatient register. BMC Public Health.

[28] Cnattingius S., Källén K., Sandström A. (2023). The Swedish medical birth register during five decades: documentation of the content and quality of the register. Eur J Epidemiol.

[29] Cheng L., Albers P., Berney D.M. (2018). Testicular cancer. Nat Rev Dis Primers.

[30] Yao S., Kuja-Halkola R., Thornton L.M. (2016). Familial liability for eating disorders and suicide attempts evidence from a population registry in Sweden. JAMA Psychiatry.

[31] Skakkebaek N.E., Rajpert-De Meyts E., Buck Louis G.M. (2016). Male reproductive disorders and fertility trends: influences of environment and genetic susceptibility. Physiol Rev.

[32] Mulhall J.P., Trost L.W., Brannigan R.E. (2018). Evaluation and management of testosterone deficiency: AUA guideline. J Urol.

[33] Sfanos K.S., De Marzo A.M. (2012). Prostate cancer and inflammation: the evidence. Histopathology.

[34] Rebello R.J., Oing C., Knudsen K.E. (2021). Prostate cancer. Nat Rev Dis Primers.

[35] Barlow L., Westergren K., Holmberg L., Tälback M. (2009). The completeness of the Swedish Cancer Register – a sample survey for year 1998. Acta Oncol.

[36] Steven L., Cherian A., Yankovic F., Mathur A., Kulkarni M., Cuckow P. (2013). Current practice in paediatric hypospadias surgery; a specialist survey. J Pediatr Urol.

