# Supplementary material for: Risk of Urological Cancer Among Boys and Men Born with Hypospadias: A Swedish Population-based Study
Source: Eur Urol Open Sci. 2023 Sep 28;57:51–9. doi: 10.1016/j.euros.2023.09.009 (PMC10658418; doi:10.1016/j.euros.2023.09.009)
Supplement: Supplementary data 1 [file mmc1.pdf]

Supplementary material

Table S1 ICD codes for hypospadias, epispadias, and cryptorchidism

ICD-7<br>1958–1968	ICD-8<br>1969–1986	ICD-9<br>1987-1996	ICD-10<br>1997-
	**Hypospadias**		
Hypospadias (757.21)	Hypospadias glandis (752.20)*	Hypospadias (752G)	Glandular hypospadias (Q54.0)*
	Hypospadias scrotalis (752.21)**		Penile hypospadias (Q54.1)*
	Hypospadias totalis (752.22)**		Penoscrotal hypospadias (Q54.2)**
	Hypospadias alia sive NUD (752.29)		Perineal hypospadias (Q54.3)**
			Hypospadias not otherwise specified (Q54.8, Q54.9)
	**Epispadias**		
757.20	752.30	***	Q64.0
	**Cryptorchidism**** **		
757.00	752.10	752F	Q53

*Grouped as distal hypospadias.
**Grouped as proximal hypospadias.
***In ICD-9, epispadias and hypospadias have the same ICD code (752G). We defined all men with that diagnosis as having hypospadias and excluded everyone who had also received a specific epispadias diagnosis in ICD-7, ICD-8, or ICD-10.
****Both diagnosis and registered radical surgical treatment of cryptorchidism (6790, KFH00) or orchidopexy (KFH10) were required.

Table S2 ICD-codes for study outcomes. The Swedish Cancer Register did not use ICD-8 to register cancers but instead continued with ICD-7 and introduced ICD-9 from 1987.

	ICD-7	ICD-9	ICD-10
**Breast cancer**	170	174	C50
**Bladder cancer**	181.0, 181.6	188	C67
**Urethral cancer**	181.2	189.3	C68.0
**Kidney cancer**	180.0, 180.9	189.0, 189.6	C64
**Other upper urinary tract cancer**	180.1, 181.1	189.1, 189.2	C65, C66
**Prostate cancer**	177	185.9	C61
**Other male reproductive cancers**	179	187	C60, C63
**Testicular cancer**	178	186.9	C62

Table S3 Specific subtyping of Wilms' tumour and testicular cancer using ICD-O codes. Anatomical codes were first used to identify kidney cancer and testicular cancer respectively, and ICD-O codes were used to further subtype.

	ICD-O/2	ICD-O/3
**Wilms' tumour**	89603	89603
**Seminoma**	9060, 9061, 9062, 9064	9060, 9061, 9062, 9064
**Seminoma strict definition**	90613	90613
**Non-seminoma**	90703, 90713, 90801, 90803, 90813, 90843, 91003	90703, 90713, 90801, 90803, 91003

Table S4 Unadjusted analyses for the association between hypospadias and testicular cancer and Wilms' respectively.

	Unadjusted HR (95% CI)		
	**Any hypospadias**	**Distal hypospadias**	**Proximal hypospadias**
**Wilms' tumor**	4.93 (2.03–12.0) [a]	NA	NA
**Testicular cancer**	2.07 (1.44–2.96)	2.14 (1.38–3.32)	12.9 (6.72–24.9) [a]
**Seminoma[b]**	2.93 (1.82–4.73) [a]	2.55 (1.37–4.76)	24.1 (10.8–53.8) [a]
**Non-seminoma[b]**	0.87 (0.36–2.10)	1.42 (0.59–3.42)	NA

[a] Hazard ratio should be interpreted as an average over the follow-up period.
[b] Analysis excluding all other testicular cancer cases from the study population

Table S5 Sensitivity analysis excluding boys and men diagnosed with cryptorchidism

	Adjusted HR (95% CI)		
	**Any hypospadias**	**Distal hypospadias**	**Proximal hypospadias**
**Testicular cancer**	1.95 (1.34–2.85)	2.26 (1.46–3.51)	10.4 (4.69–23.3)
**Seminoma[a]**	2.71 (1.63–4.51)	2.70 (1.45–5.04)	NA
**Non-seminoma[a]**	0.92 (0.38–2.22)	1.50 (0.62–3.61)	NA

[a] Analysis excluding all other testicular cancer cases from the study population

Table S6 Unadjusted analyses for the association between hypospadias and urinary tract cancer and prostate cancer in adulthood.

	Unadjusted HR (95% CI)		
	**Any hypospadias**	**Distal hypospadias**	**Proximal hypospadias**
**Bladder cancer**	1.88 (1.13–3.12)	1.90 (0.85–4.23)	NA
**Urethral cancer**	54.6 (22.2–134)	NA	NA
**Prostate cancer**	0.88 (0.64–1.21)	0.74 (0.41–1.34)	NA

Table S7 Sensitivity analysis with a stricter diagnosis of seminoma (see table S3).

	Adjusted HR (95% CI)		
	**Any hypospadias**	**Distal hypospadias**	**Proximal hypospadias**
**Seminoma**[a]	2.60 (1.56–4.32)	2.61 (1.40–4.87)	NA

[a] Analysis excluding all other testicular cancer cases from the study population

Table S8 Sensitivity analysis excluding individuals born during ICD-7 (1958-1968) and ICD-9 (1987-1996)

	Adjusted HR (95% CI)	
	**Distal hypospadias**	**Proximal hypospadias**
**Testicular cancer**	2.17 (1.35–3.49)	13.3 (5.98–29.7)
**Seminoma**[a]	2.29 (1.14–4.59)	22.2 (8.32–59.4)

Testicular cancer: including 17 individuals with distal and 6 individuals with proximal hypospadias.
Seminoma: including 8 individuals with distal and 4 individuals with proximal hypospadias.

[a] Analysis excluding all other testicular cancer cases from the study population

Table S9 Analysis with an interaction term between hypospadias status and brother status

	Adjusted HR for testicular cancer (95% CI)		
	**Hypospadias without a brother**	**Hypospadias with a brother**	**P-value for interaction**
**Full brother**	2.54 (1.65–3.91)	1.45 (0.75–2.80)	0.16
**Half brother**	1.98 (1.33–2.93)	2.41 (1.00–5.82)	0.7

Results from a Cox regression analysis model in the full cohort including an interaction term between having a full or half brother respectively (at least one) and hypospadias status.

Table S10 Unadjusted results for familial coaggregation analysis

	HR (95% CI)		
	**Father and son**	**Full brothers**	**Half brothers**
**Testicular cancer**	1.36 (0.83–2.22)	0.83 (0.36–1.93)	1.57 (0.70–3.51)

Table S11 Familial coaggregation analysis with an interaction term between hypospadias status in person A and person B.

	Adjusted HR for testicular cancer (95% CI)			
	**Only person B (relative) has hypospadias**	**Only person A has hypospadias**	**Interaction effect**	**P-value for interaction**
**Full brother**	0.39 (0.12-1.20)	1.42 (0.74-2.70)	9.33*	0.019
**Half brother**	1.61 (0.72-3.60)	1.78 (0.80-4.00)*	NA	NA
**Father-son**	1.33 (0.80-2.22)	2.42 (1.43-4.10)*	0.70	0.7

Cox regression analysis including an interaction term between hypospadias status in person A (index person) and person B (their sibling or son). No interaction effect or p-value is presented for the analysis of half brothers as there were not enough cases where both person A and B had hypospadias. Overall, the confidence intervals reflect the low number of individuals with testicular cancer in each category. As in our main analyses for familial coaggregation (figure 4), we did not find evidence that having a relative with hypospadias is associated with testicular cancer risk. However, the results for full siblings indicate that the risk of testicular cancer may be particularly high among individuals with familial hypospadias, i.e., individuals with hypospadias who also have a full sibling with hypospadias. This could indicate possible biological differences between familial and sporadic hypospadias. However, the sample size is small, and the interaction effect did not fulfill the assumption of proportional hazards across the follow-up time.

*These effect estimates did not have proportional hazards throughout the follow-up time.

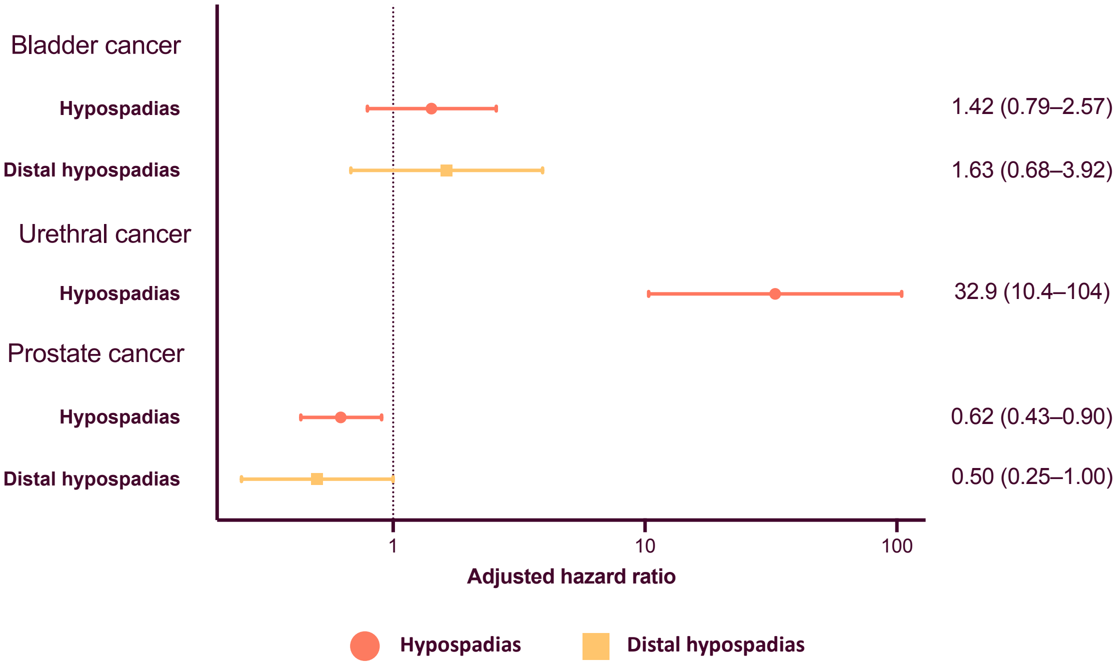

Figure S1 Sensitivity analysis excluding all individuals first diagnosed with hypospadias in the registers +/- 365 days from registered cancer diagnosis.
Bladder cancer: including 11 individuals with hypospadias and 5 individuals with distal hypospadias.
Urethral cancer: including 3 individuals with hypospadias.
Prostate cancer: including 29 individuals with hypospadias and 8 individuals with distal hypospadias.
